# Bilateral trial vocal fold injection with hyaluronic acid in patients with vocal fold atrophy with or without sulcus

**DOI:** 10.1007/s00405-019-05347-2

**Published:** 2019-03-11

**Authors:** Emke M. J. M. van den Broek, Bas J. Heijnen, Martine Hendriksma, Antonius P. M. Langeveld, Peter Paul G. van Benthem, Elisabeth V. Sjögren

**Affiliations:** Department of Otorhinolaryngology/Head and Neck Surgery, Leiden University Medical Centre, Albinusdreef 2, PO-Box 9600, 2300 RC Leiden, The Netherlands

**Keywords:** Glottic insufficiency, Vocal fold atrophy, Sulcus, Bilateral (trial) vocal fold injection, Injection laryngoplasty

## Abstract

**Purpose:**

To evaluate the outcome of bilateral trial vocal fold injection (VFI) with hyaluronic acid in patients with vocal fold atrophy ± sulcus and to assess the predictive value of trial VFI on the outcome of durable medialization procedure.

**Methods:**

Voice data collected according to a standardized protocol before and one month after trial VFI of 68 patients with vocal fold atrophy (30) and atrophy with sulcus (38) were analyzed. Voice Handicap Index (VHI)-30 was compared to the outcome of a durable medialization at 3 and 12 months.

**Results:**

The overall VHI-30 improvement was 16.8 points (from 49.9 to 33.1), which was statistically significant and clinically relevant. 57.8% of the patients experienced enough subjective benefit after trial VFI to undergo durable medialization. Of the patients that experienced subjective benefit 62% had a clinically relevant improvement in VHI-30. There was no relevant change in other parameters and no difference between ± sulcus. After durable medialization 90–94% of the patients had VHI-30 scores similar to or better than post-trial VFI.

**Conclusion:**

The majority of patients experience subjective improvement after bilateral trial VFI indicating that medialization is a valid treatment option for patients with vocal fold atrophy ± sulcus. The VHI-30 only partially overlaps with patients’ subjective evaluation and does not predict which patients will experience subjective improvement. It is, however, predictive for VHI-30 outcome after durable medialization. The aerodynamic and acoustic parameters showed no relevant change. Further identification of voice assessment parameters accurately reflecting the subjective experience of these patients is warranted.

## Introduction

Vocal fold atrophy is defined as loss of muscle bulk and tone of the thyro-aytenoid/lateral cricoarytenoid complex in a vocal fold with a normal range of motion [1]. It is a common cause of dysphonia in non-paralytic glottic insufficiency. There are several forms of vocal fold atrophy. (1) In presbyphonia there is atrophy of both the lamina propria and the vocal fold muscles, and degeneration of the cartilaginous framework due to the aging process [2]. Because of the growth of the elderly population this problem is seen more frequent in daily laryngological practice [2–4]. (2) Atrophy can also be found in younger patients who report similar complaints from childhood or early adolescence and have a comparable clinical presentation, suggesting a young adolescent form of vocal fold atrophy. This phenomenon has been observed by others [5]. Finally, (3) atrophy can also be associated with congenital forms of vocal fold scar such as sulcus [6]. In these patients, in our experience, there is often loss of muscle bulk in addition to the abnormalities found in the upper vibratory layers of the vocal fold. The treatment options for improving glottic closure in patients with vocal fold atrophy are speech therapy and/or medialization of the vocal folds. For presbyphonia improvement in vocal function after speech therapy has been observed, but is influenced by degree of atrophy, glottis closure pattern and patient’s burden of medical problems [7].

Medialization can be achieved either by bilateral vocal fold injection (VFI) with a durable injectable such as autologous fat or calcium hydroxyapatite, or by bilateral medialization thyroplasty. In patients with additional vocal fold scar there is also the option of microphonosurgery on the upper vibratory layers of the vocal fold. A recent consensus report on vocal fold scar by the European Laryngological Society (ELS) presents an overview of treatment options for these patients. These include classic medialization and epithelium freeing techniques as well as novel approaches such as the use of angiolytic lasers to soften the scar tissue, tissue engineering, and stem cell techniques. Although no definite recommendations are made, because of the unpredictability of the results, the advice of the consensus committee is to start with the least traumatizing procedure, which is usually VFI [6].

The results of treatment of vocal fold atrophy with or without vocal fold scar are less predictable than those of glottic insufficiencies caused by hypomobility or paresis [8]. Therefore, trial VFI with a short acting substance can be used to predict the outcome of a durable medialization procedure [1].

Since 2012 we have been performing trial VFI using hyaluronic acid (HA) in patients with vocal fold atrophy. Due to the varying and sometimes disappointing results of microphonosurgery in patients with vocal fold scar, trial VFI has been our first approach in this patient cohort as well. Depending on the degree of subjective benefit after trial VFI, through a shared decision making process, it is decided whether or not to move on to a durable medialization procedure. For this we prefer bilateral VFI with autologous fat or bilateral medialization thyroplasty depending on patient and/or surgeon’s preference. Our treatment strategy is in line with the algorithm for treatment for scar and glottic incompetence as proposed by Carroll et al. [9].

The purpose of this study was to evaluate the outcome of bilateral trial VFI with HA in our patients with vocal fold atrophy with or without sulcus and to assess the predictive value of trial VFI on the outcome of durable medialization procedure.

## Methods

### Patients

**Fig. 1:**
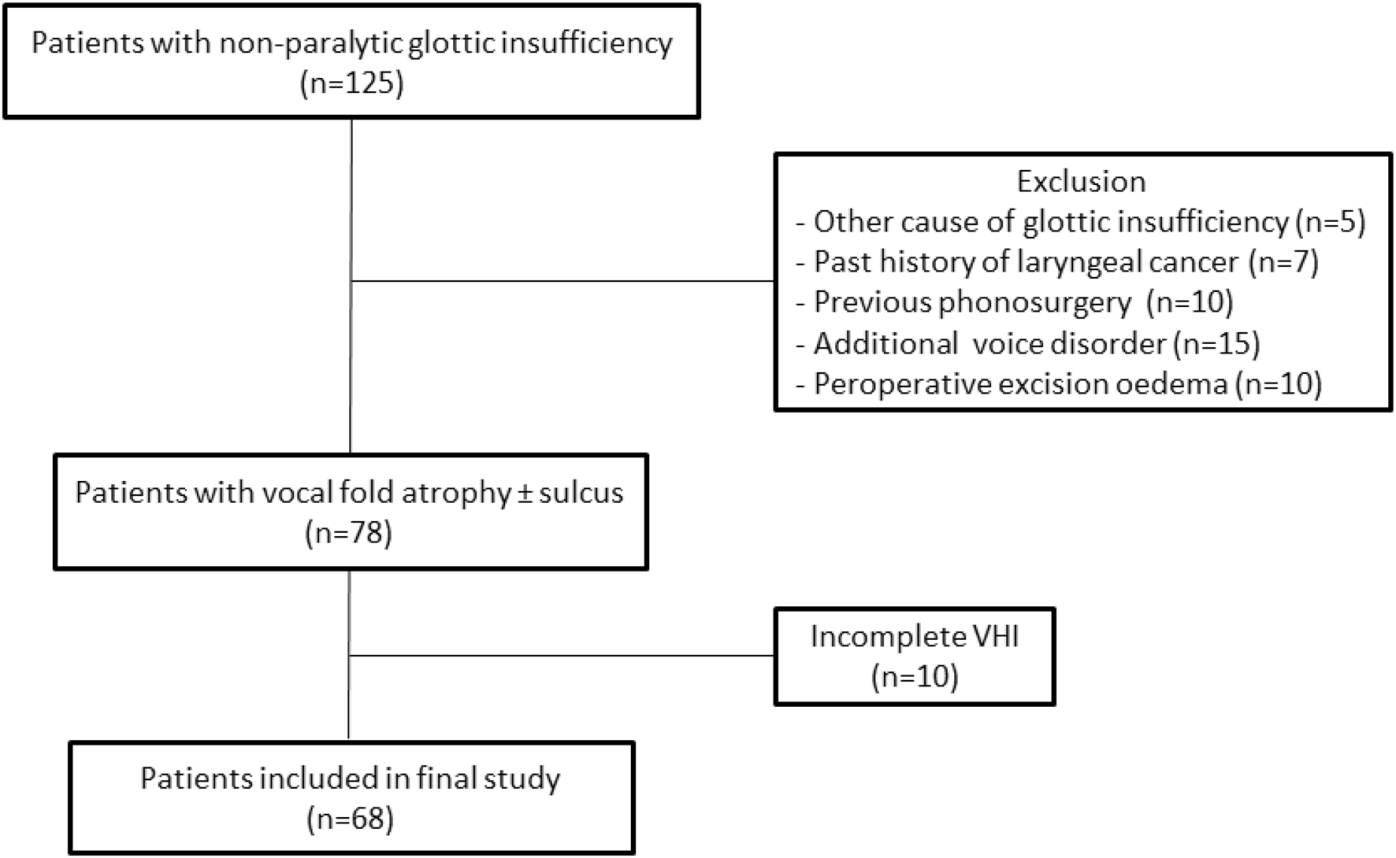
Patient selection, inclusion and exclusion criteria

Institutional review board approval for the publication of this data was obtained from the Leiden University Medical Center Ethics Committee. All patients with non-paralytic glottic insufficiency who underwent bilateral trial VFI with HA under general anesthesia (*n* = 121) or as an in-office procedure (*n* = 4) from September 2011 to April 2017 (*n* = 125) were retrospectively reviewed (Fig. 1). 47 patients were excluded because of another cause of glottic insufficiency (e.g., paresis or glottic insufficiency post intubation) (*n* = 5), a past history of laryngeal cancer (*n* = 7), previous phonosurgery (*n* = 10), an additional voice disorder (e.g., vocal tremor, cyst) (*n* = 15) or peroperative excision of oedema (*n* = 10). The remaining 78 patients had been diagnosed with vocal atrophy based on the clinical features of glottic incompetence (atrophic appearance of the vocal folds with or without sulcus while retaining a normal range of motion). Of these 78 remaining patients, 68 had pre- and postoperative voice data with at least a complete Voice Handicap Index (VHI)-30 questionnaire and were included in the definitive analysis. These 68 patients had undergone trial VFI under general anesthesia between June 2012 and April 2017. Patients routinely started speech therapy within a week of the injection and continued as long as deemed necessary by the speech-language therapist. The outcome was evaluated based on two criteria: (1) voice analysis data collected routinely at one month after trial VFI and (2) patients’ subjective perception of benefit during their return visit at 3 months after trial VFI. If the voice analysis data did not match the patient’s own appraisal, the patient’s subjective perception was leading both in determining the final result of the trial VFI and the decision regarding a durable medialization procedure. Therefore, patients that experienced enough subjective benefit to be motivated for a durable medialization procedure were considered good responders.

### Voice data

Voice outcome data were collected according to a standardized voice analysis protocol implemented preoperatively and at 1 month postoperatively. This protocol included patients’ self-assessment using the VHI-30, aerodynamic evaluation with maximum phonation time (MPT) and dynamic range and acoustic analyses including fundamental frequency (F0) and melodic range. Voice outcome data for the durable medialization procedure were collected using the same protocol preoperatively as well as 3 and 12 months postoperatively. In this study the VHI-30 was the primary outcome parameter of the voice analysis protocol. It is a patient-based self-assessment tool consisting of 30 items, which are distributed over three domains: functional, physical, and emotional [10]. In the Dutch version of the VHI-30 a score of 15 points or more identifies patients with voice problems in daily life [11, 12]. Furthermore, a change in pre- and postoperative score of 10 points or more in the individual patient and 15 points or more for a group can be considered clinically relevant [12]. The MPT was measured on/a/at comfortable pitch and loudness. The longest MPT from two attempts was included for analysis. Fundamental frequency in hertz (Hz), dynamic range in decibel (dB) and melodic range in semitones (ST) were extracted from the patient’s phonetogram recorded with the voice profiler (Alphatron, Rotterdam, the Netherlands, 2007) in standardized settings.

### Procedure

All procedures were performed by an experienced laryngologist and/or a fellowship trained laryngologist. The trial VFI was performed in general anesthesia as this allows for the palpation of the vocal folds to distinguish between atrophy and atrophy with sulcus. Also, the extent of a sulcus can be assessed as part of the work-up for possible future microphonosurgery with sulcus excision. In our study sulcus was defined as a pathological type 2 sulcus and/or type 3 sulcus according to the Ford classification [13]. This corresponds to a sulcus vergeture and/or sulcus vocalis in the classification by Bouchayer [14]. The material used for the trial VFI was hyaluronic acid (Juvéderm^®^ ULTRA SMILE, Allergan, Dublin, Ireland). The injection was bilateral in all cases. The standard practice was to inject in the lateral part of the thyroarytenoid muscle at the level of the vocal fold process. If necessary a second injection point was chosen at the level of the midcord. Injection was continued until adequate medialization was achieved according to the clinical experience of the surgeon. Typically 0.15–0.25 cc per vocal fold was used. Durable VFI was performed in general anesthesia using autologous fat harvested by abdominal liposuction. Bilateral medialization thyroplasty was performed in local anesthesia using Gore-tex^®^ (GORE-TEX^®^ Soft Tissue Patch, Gore Medical, Flagstaff, Arizona) as implant material as described by Isshiki and McCulloch with some modifications [15, 16].

### Statistical analysis

All data were analyzed using SPSS for windows (IBM SPSS Statistics for Windows, Version 21.0, released 2012. IBM Corp, Armonk, NY). Data showed a normal distribution. The pre- and postoperative voice data for the overall patient group were analyzed using a linear mixed model. A linear regression model was used to analyze the association between various factors (age, gender, and pathology) and voice outcome. The Chi-squared test was used to compare the dichotomous variables “clinically relevant VHI improvement” and “durable medialization”. For all statistical tests a *p* value smaller than 0.05 was considered significant.

## Results

Demographic details at baseline of the 68 patients with vocal fold atrophy (*n* = 30) or atrophy with sulcus (*n* = 38) that underwent bilateral trial VFI with HA and the type of durable medialization according to patients’ choice are shown in Table 1. 27 did not experience enough subjective benefit to want to undergo a durable medialization procedure. Three of these were patients with sulcus who elected to undergo microphonosurgery (data not shown).

**Table 1:** Demographic details of patients with vocal fold atrophy ± sulcus who underwent bilateral trial vocal fold injection and their choice of durable medialization

Characteristics	Total	Atrophy	Atrophy with sulcus
	*n* = 68 (100%)	*n* = 30 (44.1%)	*n* = 38 (55.9%)
Trial VFI			
Mean age, baseline (SD)	40 (18.5)	42 (19.5)	39 (17.7)
Gender (%)			
Male	18 (26.5)	7 (23.3)	11 (28.9)
Female	50 (73.5)	23 (76.7)	27 (71.1)
Postoperative voice outcome timing (%)			
At 1 month	53 (77.9)	24 (80)	29 (76.3)
At other time (range 2–8 weeks)	15 (22.1)	6 (20)	9 (23.7)
Durable medialization			
Bilateral VFI with autologous fat	19 (27.9)	8 (26.7)	11 (28.9)
Bilateral medialization thyroplasty	18 (26.5)	10 (33.3)	8 (21.1)
No durable medialization procedure	27 (39.7)	10 (33.3)	17 (44.7)
Undecided	4 (5.9)	2 (6.7)	2 (5.3)

*VFI* vocal fold injection, *SD* standard deviation

The pre- and post-injection voice outcome data for the overall patient group are shown in Table 2. The mean VHI-30 for the overall group decreased by 16.8 points from 49.9 to 33.1. This improvement was both statistically significant and clinically relevant (improvement ≥ 15 points). Melodic range improved from 16.5 to 19.6 ST, which was also statistically significant. For the other voice parameters changes in pre- and post-trial outcomes were not statistically significant.

**Table 2:** Pre-and post-trial vocal fold injection voice outcome data of patients with vocal fold atrophy ± sulcus

Voice outcome	Mean pre-trial VFI value (95% CI)	Mean post-trial VFI value (95% CI)	Mean difference (95% CI)	*p* value
VHI-30	49.9 (45.6; 54.2)	33.1 (28.8; 34.4)	− 16.8 (− 21.5; − 12.1)	< 0.001*
MPT (s)	12.4 (10.8; 14.0)	12.5 (10.9; 14.1)	0.1 (− 1.5; 1.6)	0.932
*F*_0_ (Hz) male	158 (140; 176)	149 (131; 167)	− 9 (− 21; 3)	0.135
*F*_0_ (Hz) female	202 (192; 212)	205 (195; 215)	3 (− 3; 9)	0.358
Dynamic range (dB)	30.1 (27.2; 33.1)	32.9 (29.9; 35.9)	2.7 (− 0.2; 5.6)	0.069
Melodic range (ST)	16.5 (14.5; 18.6)	19.6 (17.5; 21.6)	3.0 (1.1; 4.9)	0.002*

*VFI* vocal fold injection, *CI* confidence interval, *VHI* voice handicap index, *MPT* maximum phonation time, *F*_*0*_ fundamental frequency, *Hz* hertz, *dB* decibel, *ST* semitone

*p value < 0.05 was considered significant

Table 3 shows the influence of various patient factors on voice outcome after trial VFI with HA in a univariate linear regression model. There was no association between gender or sulcus and the post-trial change in any of the voice parameters. For age a significant association was found with two of the five voice parameters showing an improvement in outcome with increasing age (VHI-30 and dynamic range).

**Table 3:** Patient factors age, gender and pathology, and their influence on voice outcome parameters

Covariance	Age (continue)	Gender (male/female)	Pathology (vocal fold atrophy ± sulcus)
	*B* (95% CI), *p* value	*B* (95% CI), *p* value	*B* (95% CI), *p* value
VHI-30	− 0.301 (− 0.55; − 0.05), 0.018*	1.156 (− 9.6; 11.9), 0.830	4.663 (− 4.8; 14.1), 0.329
MPT	0.056 (− 0.03; 0.14), 0.183	0.146 (− 3.4; 3.7), 0.934	− 1.094 (− 4.2; 2.0), 0.482
*F*_0_ male	0.137 (− 0.66; 0.94), 0.720	NA	− 5.106 (− 11.7; 1.5), 0.120
*F*_0_ female	− 0.473 (− 1.1; 0.11), 0.109	NA	0.528 (− 4.3; 5.4), 0.828
Dynamic range	0.169 (0.01; 0.32), 0.033*	2.111 (− 4.5; 8.7), 0.524	− 4.009 (− 9.8; 1.8), 0.174
Melodic range	− 0.954 (− 4.9; 2.9), 0.62	− 0.710 (− 5.1; 3.6), 0.745	0.058 (− 0.05; 0.16). 0.277

*B* beta coefficient, *CI* confidence interval, *VHI* voice handicap index, *MPT* maximum phonation time, *F*_0_ fundamental frequency, *NA* not applicable

*p value < 0.05 was considered significant

40 of the 68 patients (58.8%) had a clinically relevant improvement in their VHI-30 score after trial VFI (improvement ≥ 10 points). Their mean improvement was 29.1 points (SD 14.6). 28 patients (41.2%) did not have a clinically relevant improvement (improvement < 10 points or a deterioration of the VHI-30 score). Their mean improvement was − 0.8 points (SD 8.8) (data not shown).

Table 4 shows clinically relevant VHI-30 improvement (yes/no) according to patients’ choice regarding a durable medialization procedure (yes/no). There was only a partial overlap between these two parameters. Of the 37 patients that chose to undergo a durable medialization procedure only 23 (62.2%) had a clinically relevant improvement in their VHI-30 score. This means that 14 patients (37.8%) chose to undergo a durable medialization procedure because they experienced a subjective benefit of the trial VFI that was not reflected in their VHI-30 score. 15 (55.6%) of the 27 patients that did not chose to continue to a durable medialization procedure because they did not experience enough subjective benefit still had a clinically relevant improvement in their VHI-30 score. The reasons they did not want to continue to a durable procedure was prolonged improvement in voice outcome after the bilateral trial VFI (*n* = 6), lack of motivation to undergo a second procedure (*n* = 5) or no self-reported improvement after the trial VFI despite a clinically relevant improvement in VHI-30 score (*n* = 4). This pattern of partial overlap was seen in both patients with and without sulcus. Statistically, there was no significant difference in the proportions between these subgroups (data not shown).

**Table 4:** Patients’ choice for durable medialization procedure according to clinically relevant VHI-30 improvement

Durable medialization	Total = 64	
	Yes = 37 (57.8%)	No = 27 (42.2%)
Clinically relevant VHI-30 improvement^a^		
Yes	23 (62.2)^b^	15 (55.6)
No	14 (37.8)	12 (44.4)

^a^VHI-30 improvement ≥ 10 points in individual patient

^b^No sign. difference was found for clinically relevant VHI-30 improvement and durable medialization (*p* = 0.595)

As stated earlier 37 patients initially chose to have a durable medialization procedure, of which 31 have been carried out to date (5 patients have had second thoughts and canceled their procedure for unknown reasons and 1 patient is still on the waiting list). Of the 31 patients that have had a durable medialization procedure 2 were lost to follow-up between 0 and 3 months. Therefore, voice analyses data were available for 29 patients at 3 months after long-term medialization. At the 12-month interval voice analyses were available for 18 of the 31 patients; 3 patients were lost to follow-up between 3 and 12 months, 1 patient underwent revision thyroplasty within 12 months and 9 patients were still short for their 12 month follow-up period. The mean VHI-30 score was 34.2 (± 16.5) at 3 months and 25.2 (± 14.6) at 12 months after the durable procedure.

**Fig. 2:**
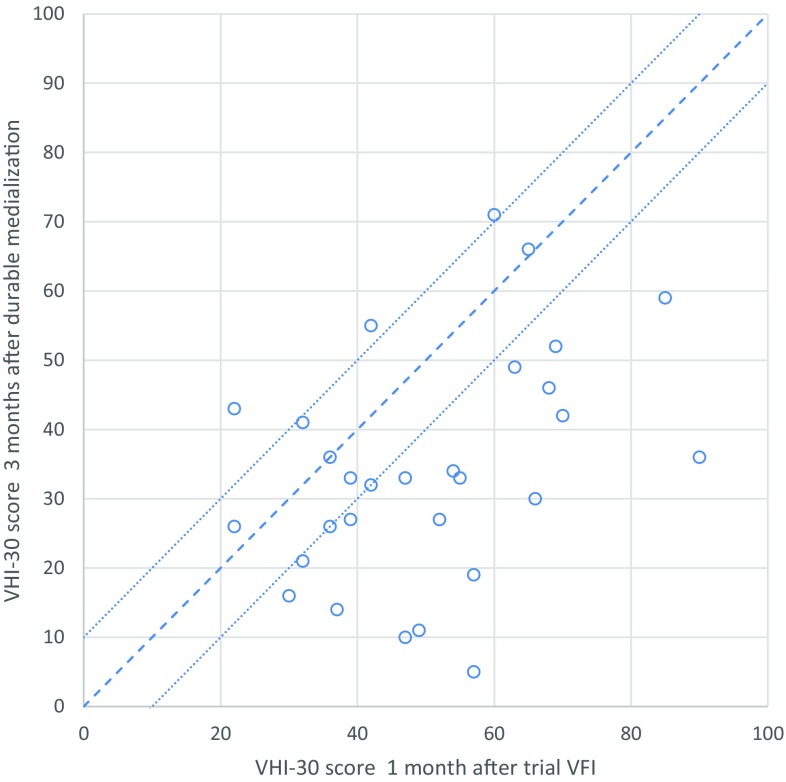
VHI-30 score 3 months after durable medialization compared to VHI-30 score 1 month after bilateral trial vocal fold injection (*n* = 29). *VHI* voice handicap index, *VFI* vocal fold injection

Figure 2 shows the individual VHI-30 score 1 month after bilateral trial VFI plotted against the VHI-30 score 3 months after durable medialization (bilateral VFI with autologous fat *n* = 11; bilateral medialization thyroplasty *n* = 18). In 26 patients (90%) the VHI-30 score after the durable procedure was either comparable to (within a 10-point range, *n* = 7) or better than (> 10 points improved, *n* = 19) the VHI-30 score after trial injection. Three patients had a deterioration of more than 10 points in their VHI-30 scores 3 months after durable medialization procedure compared to the trial VFI.

**Fig. 3:**
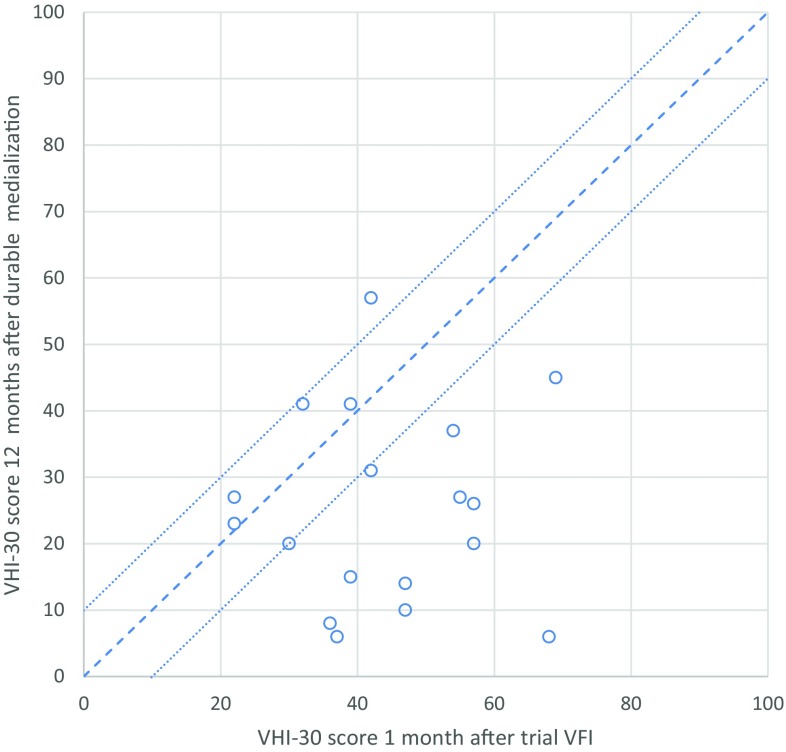
VHI-30 score 12 months after durable medialization compared to VHI-30 score 1 month after bilateral trial vocal fold injection (*n* = 18). *VHI* voice handicap index, *VFI* vocal fold injection

Figure 3 shows the individual VHI-30 score 1 month after trial VFI plotted against the VHI-30 score 12 months after durable medialization (bilateral VFI with autologous fat *n* = 11; bilateral medialization thyroplasty *n* = 7). In 17 patients (94%) the VHI-30 score for the durable procedure was either comparable to (within a 10-point range, *n* = 5) or better than (> 10 points improved, *n* = 12) the VHI-30 score after bilateral trial VFI. One patient had a deterioration of 15 points in VHI-30 12 months after durable procedure compared to the trial VFI.

## Discussion

In this study we evaluated the outcome of bilateral trial VFI with HA in 68 patients with vocal fold atrophy [atrophy only (*n* = 30) and atrophy with sulcus (*n* = 38)] and assessed the predictive value of trial VFI on the outcome of a durable medialization procedure. To our knowledge this is the largest study on trial VFI to date.

We found that the majority of patients reported a good response to the trial VFI, which was defined as enough subjective benefit to want to undergo a durable medialization procedure. The proportion of patients with a good response was not influenced by the type of pathology. We also found a statistically significant and clinically relevant improvement in the overall VHI-30 score. Looking at individual patients, the majority (58.8%) showed a clinically relevant improvement in the VHI-30.

These results demonstrate that the majority of patients with atrophy with or without sulcus experience less voice handicap after trial VFI, indicating that medialization is a valid treatment option in both these patient groups. However, the group of patients that chose to undergo a durable medialization procedure only partially corresponded to the group with a clinically relevant improvement in the VHI-30. Therefore, our findings show that even though the VHI-30 is indicative for voice improvement in this overall patient group, it does not adequately identify the subgroup of “true” good responders who will be motivated for a durable medialization procedure based on their subjective benefit of the trial VFI.

The concept of trial vocal fold injection was defined in 2010 [1]. Since then at least five studies have looked at the change in voice parameters after trial injection, with study populations ranging from 19 to 35 patients [1, 9, 17–19]. The study by Young et al. was done in patients with atrophy, excluding patients with hypomobility, paresis or paralysis [17]. The other studies did include patients with hypomobility or paresis. One study also included one patient with a vocal fold paralysis [19]. Our proportion of patients with a good subjective response (57.8%) is comparable with those found by Carroll. He found a good response in 32–75% of patients in his three studies [1, 9, 19]. When including partial responses this proportion increased to 76–83% [1, 9, 19]. As for the VHI, it is not possible to compare our findings directly to those of other studies as they used either the VHI-10 version [1, 17, 19] or an alternative self-assessment tool [18]. However, when considering the proportion of patients with a clinically relevant change in the VHI in the different studies, our study (58.8%) has a relatively high percentage compared to earlier reports by Young (42%) and Carroll (30.4%) [17, 19]. Although the VHI scores improve it is important to realize that all studies show that the VHI does not normalize after trial VFI. Even the subgroups of good responders show elevated scores indicating an on-going burden on voice use in these patients. In our study good responders had a mean post-injection VHI-30 score of 26.7 (normal value VHI-30 Dutch version < 15) [12]. The mean post-injection VHI-10 score in good responders in the study by Young et al. was 12 and 20.5 in the study by Carroll et al. [17, 19] (normal value VHI-10 English version ≤ 11) [20].

Finally, the discrepancy that we found between the patients’ subjective experience and the VHI outcome has also been noted by others; a recent study showed that although 65% of the patients reported a subjective good response only five out of 15 (33%) had a clinically relevant improvement in their VHI-10 score (≥ 5 points improvement) [19]. The value of the VHI for assessing voice improvement in patients with glottic incompetence due to atrophy with or without sulcus is, therefore, unclear and needs to be further explored.

As for the acoustic and aerodynamic parameters, we found a statistically significant improvement in the melodic range from 16.5 to 19.6 ST. However, this would still classify as a limited range according to values for healthy subjects found by Wuyts. In this publication healthy subjects showed a melodic range between 22 and 44 ST [21]. Also, it is unclear what the clinical relevance of this increase in melodic range would actually mean to the patients. We found no statistically significant changes in the other voice parameters. The limited effect seen in acoustic and aerodynamic parameters in our study are reflected in other studies [17, 19].

As the effect of bilateral trial VFI is not reflected in the objective voice measures as described above and is only partially reflected in a clinically relevant VHI improvement two questions arise: (1) are the voice parameters used in these publications sufficiently valid and precise and (2) is the timing of the voice data acquisition optimal? The VHI is proven to be a reliable tool for measuring the general impact of a voice disorder and patient burden [10]. Also, as vocal effort is reflected in aerodynamic parameters, we expected these to change after trial VFI. However, some authors are currently pointing out that disease-specific outcome tools may be needed to accurately reflect voice impairment and to measure outcome of intervention [22, 23], which is in line with our findings. Alternative voice outcome measurements that better represent the voice problems of this patient group need to be identified. Two questionnaires for analyzing subjective voice change in patients with glottal insufficiency are mentioned in the literature: the glottal function index (GFI) and the vocal fatique index (VFI) [24, 25]. The GFI is a four-item symptom index, which can be used in the evaluation and treatment in patients with glottal dysfunction [24]. Although the questionnaire has not been designed for a specific clinical disease entity it has been used in several studies on non-paralytic glottic incompetence and its treatment [8, 18, 26, 27]. The VFI is a questionnaire designed specifically for detecting vocal fold fatigue and assessing the impact of treatment these patients [25].

These questionnaires could be of added value when assessing subjective voice change in patients with glottic incompetence, but validated Dutch versions have not yet been developed. Alternative aerodynamic parameters more suited for atrophy patients could be the phonation threshold pressure (PTP) and phonation threshold flow (PTF) reflecting the minimum pressure and flow required to sustain phonation. These parameters may better mirror the ease of phonation [28] [personal communication S. Thibeault and D. Phyland]. Frame-by-frame analysis (FBFA) of laryngovideostroboscopy in which subject’s average percentage of closed frames per glottic cycle is recorded may be another option [29]. The use of 2D digital kymography using laryngeal high-speed video-endoscopy allowed Bae to identify vibratory changes after VFI in patients with paresis and after speech therapy in patients with atrophy [30]. The benefit of these and other alternative voice outcome parameters as well as their clinical implications need to be further explored for patients with atrophy with or without sulcus. For now, we agree with the decision to continue to a definitive procedure is mainly based on the patient’s subjective experience and that additional voice data are of limited contribution [1].

Next to selecting suitable voice outcome parameters, the timing of evaluation is of great importance when evaluating the success of trial VFI. In our study post-trial injection data were collected at 1 month, with a spread from 2 to 8 weeks for a minority of patients. As the different substances used in the studies described have different lifespans it is difficult to compare results, although these other studies also raise concerns about the optimal timing of data collection. In our experience the window of benefit varies per patient even if the same substance is used and depends on factors such as the amount injected, possible oedema or temporary stiffness of the vocal folds and patient compliance with speech therapy. Due to these variations we assume that the voice outcome data in our study will not have been collected at the optimal time point in all patients. Interestingly some of the patients reported noticing a subjective benefit only after the effect of the injection had worn off. The confrontation with their “old” voice made them realize that the injection had been helpful. This subjective effect will, therefore, not have been captured in the VHI-30 filled in during voice analysis at 1 month. Ideally, evaluation should be done frequently to capture voice data at the time of patients’ optimal benefit from the trial VFI. However, for practical reasons we chose to evaluate at 1 month as (1) patients in our experience generally undergo an adjustment period the first few weeks when the voice is not optimal and (2) HA has an average lifespan of 3 months. We do agree that further studies are warranted determining the optimal timing for data collecting post-trial injection [19].

In our study we found that neither gender, nor the additional presence of a sulcus had any influence on voice outcome. We did find an association between age and two of the five voice parameters (VHI and dynamic range) showing an improvement in their outcome with increasing age. This influence of age has not been reported before. We hypothesized that a lower voice demand in the absence of work-related voice use may be underlying cause for increased VHI improvement with increasing age. In the literature, although the data are limited, poorer results after medialization procedures in patients with vocal fold scar have been reported [8, 27]. This poorer result may be well explained by the “double” pathology, not only affecting the glottic closure, but also affecting the vibratory potential of the layered anatomy of the vocal fold. However, although we conceptually agree with this assumption, these findings were not confirmed in our study for patients with sulcus undergoing trial VFI with HA. Our results may have been influenced by the fact that we included only patients with sulcus (Ford type II and III), which is a specific form of vocal fold scar. Further studies on patients with vocal fold scar and specifically with sulcus are needed to determine the value of (trial) medialization in this subcategory.

In this study the VHI-30 outcome did not always correspond to the patients’ subjective experience of the trial VFI. However, we did demonstrate the ability of the VHI-30 outcome after trial VFI to predict the VHI-30 outcome of a durable medialization procedure. 3 months after durable medialization procedure 90% of patients had a similar or better VHI-30 score compared to after trial VFI. This increased to 94% at 12 months. This is in line with previous studies that also have found a predictive value of trial VFI. In 2010 Carroll and Rosen described that all patients with a good subjective result after their trial VFI also had a good result after the durable medialization procedure [1]. In the study by Young et al., 75% of the patients with a good response to trial VFI (VHI-10 improvement of ≥ 5 points) also had a good response to the durable medialization procedure. 55% of patients with a poor response to trial VFI also had a poor response after the durable medialization procedure. Conversely 45% of patients had a good response after the durable procedure despite failing the trial VFI, indicating that the predictive value of a failed trial VFI might be lower than a successful one [17]. As theirs is the only study in which all patients with trial VFI underwent a durable medialization procedure this information cannot be compared to other studies. Interestingly, a recent study also showed the predictive value for trial VFI in patients with vocal fold scar. In this study 80% of good responders to trial VFI also showed clinically significant improvement after durable augmentation [9]. Dumberger et al., reported on predictability of trial VFI in 35 patients with non-paralytic glottic incompetency, including patients with paresis, who underwent trial VFI followed by type 1 medialization with Gore-Tex. They concluded that trial VFI is a useful tool, showing a good correlation (*r* = 0.55) with change in VRQOL, a strong correlation with change in GFI (*r* = 0.74) and excellent correlation with change in GRBAS (*r* = 0.90) [18].

Our study had some limitations. First, although the data were collected using a standardized protocol at set time points as a part of routine voice outcome registration, the study design was retrospective. This possibly led to more missing data than if the study had been prospective. We corrected for this where possible using a linear mixed model for analysis of change in pre- and post-trial VFI voice data. Additionally, there were missing data at the 12-month interval after the durable medialization procedure, mainly because nine patients were still not 12 months out from their operation. Second, the size of the patient group is a limitation. Although with 68 patients it is the largest series in this patient group to date, the isolated atrophy (*n* = 30) and atrophy with sulcus groups (*n* = 38) are still relatively small. A third limitation is that two different long-term procedures were used. It is still unclear which durable medialization procedure is the most beneficial for this patients group. We consider the current sample size too small to investigate the difference between the two procedures and this question was also beyond the scope of this study. Also, we did not investigate a possible difference for patients with atrophy versus atrophy with sulcus for the long-term procedure. Furthermore, the definition of a good responder in this study was based on the patient’s motivation to continue to a durable medialization procedure, which was in turn based on their subjective experience of the trial VFI. We recognize that there were five patients who initially opted for a durable procedure who had second thoughts and chose to cancel their procedure. As we had thorough discussions with each patient before consenting them for a long-term procedure, to make sure that the effect of the trial VFI was sufficient, and as we do not know the reason for their cancelation we have included them as good responders in analysis. Furthermore, a few patients with a good response did not opt to continue to a permanent procedure. Therefore, our estimation of good responders may be slightly over or under valued. We do not expect this deviation to be significant as in the group of poor responders the joint decision was always to refrain from a durable medialization procedure. Finally, we already mentioned our concerns about validity of the voice outcome parameters used and the timing of data collection. Further studies and consensus guidelines on the optimal voice outcome parameters for this patient group are warranted.

## Conclusion

This study shows that a majority of patients (57.8% in this study) with non-paralytic glottic incompetence due to atrophy with or without sulcus experienced subjective improvement in voice after trial VFI. Patients showed a statistically significant and clinically relevant improvement in overall VHI-30 score. This indicates that vocal fold medialization is a valid treatment option in patients with vocal fold atrophy and atrophy with sulcus. However, the VHI-30 score and patients’ subjective experience only partially overlapped and the VHI-30 is, therefore, not able to adequately predict patients’ subjective experience. However, in patients with a good response to trial VFI who choose to undergo a durable medialization procedure the VHI-30 score is predictive for a similar or better VHI-30 outcome after durable medialization procedure. Aerodynamic and acoustic parameters in our study showed no relevant change. Therefore, in line with previous publications, this study suggests that the general voice parameters used may not be suitable for this patient population and further exploration to find specific voice assessment tools is necessary. To our knowledge this is the largest study on trial VFI to date.
